# Efficiency of Repetitive Transcranial Direct Current Stimulation of the Dorsolateral Prefrontal Cortex in Disorders of Consciousness: A Randomized Sham-Controlled Study

**DOI:** 10.1155/2019/7089543

**Published:** 2019-06-11

**Authors:** Min Wu, Yamei Yu, Lunjie Luo, Yuehao Wu, Jian Gao, Xiangming Ye, Benyan Luo

**Affiliations:** ^1^Department of Neurology & Brain Medical Centre, First Affiliated Hospital, School of Medicine, Zhejiang University, Hangzhou 310003, China; ^2^Department of Rehabilitation, Hangzhou Hospital of Zhejiang Armed Police Corps, Hangzhou 310051, China; ^3^Department of Rehabilitation Medicine, Zhejiang Provincial People's Hospital, People's Hospital of Hangzhou Medical College, 310014, China

## Abstract

Conventional transcranial direct current stimulation (tDCS) targeting the left dorsolateral prefrontal cortex (DLPFC) could improve arousal in disorders of consciousness (DOC). However, the comparative effectiveness of anodal stimulation of the left DLPFC and the electrophysiological effect of tDCS are yet to be determined. In this randomized sham-controlled design, patients were separated into three groups (left/right anodal tDCS, sham). Data on the clinical assessments and EEG were collected at baseline and after 2 weeks of tDCS. The outcome at 3-month follow-up was evaluated using the Glasgow Outcome Scale-Extended. Results showed that sessions of the left tDCS facilitated the excitability of the prefrontal cortex, whereas only one patient had a positive outcome. Targeting the right DLPFC was less effective, merely leading to activation of the stimulation site, with no effect on the state of arousal. Moreover, sham stimulation had minimal or no effect on any of the outcomes. These results provide evidence for a hemispheric asymmetry of tDCS effects in patients with DOC. Left anodal tDCS might be more effective for modulating cortical excitability compared to tDCS on the right DLPFC. However, future studies with large sample sizes are needed to confirm these findings. This trial is registered with NCT03809936.

## 1. Introduction

Parallel to the growing demand for an effective noninvasive approach with neuromodulatory potential, interest in transcranial direct current stimulation (tDCS) has increased. Recently, numerous clinical studies have reported that tDCS ameliorates several clinical conditions and improves functions ranging from sensation to cognition in healthy subjects and patients with neurological or psychiatric disorders [1–4]. These promising findings have also led to some tDCS protocols in patients with disorders of consciousness (DOC) after severe brain injury, where encouraging results were found in a number of patients with minimally conscious state (MCS) by particularly targeting the left dorsolateral prefrontal cortex (DLPFC) [5].

Most of these studies only performed subjective behavioral analyses, and although they are encouraging from a clinical point of view, the extent of tDCS-mediated effects on brain physiology remains largely elusive and requires further investigation. A recent cross-over study investigated cerebral activity using electroencephalography (EEG) visual analysis, which might fail to detect minor and focal cerebral changes [6]. Bai et al. used TMS-EEG to assess cortical excitability changes after a single session of anodal tDCS and showed preliminary evidence that tDCS could effectively modulate cortical excitability in patients with DOC [7]. Another study demonstrated behavioral effects of 4-week tDCS, which can moderately improve recovery of patients in MCS [8]. It is thus conceivable that repeated tDCS sessions could induce preferable effects. However, to date, the EEG efficiency of long duration tDCS in DOC is limited.

Given that tDCS relies on electrical current flowing from an anode to a cathode, it can be difficult to ascribe observed behavioral effects of tDCS to specific stimulation sites [9]. Instead, it has been hypothesized that tDCS acts by modulating functional connectivity [10, 11]. Previous studies using magnetic resonance imaging (MRI) and EEG to investigate the effects of prefrontal tDCS on functional connectivity found significant changes in the widespread brain connectivity of healthy subjects [12, 13]. Nonetheless, few studies have explored the regulatory mechanisms at the network level in specific DOC patients, which are characterized as having a complex dysfunctional connectivity pattern [14]. To investigate these issues, we performed tDCS combined with electrophysiological techniques to evaluate neuroconnectivity modulation in patients with DOC.

Another critical issue is the lack of evidence on whether the left DLPFC is the most optimal target for tDCS. Among the existing studies involving tDCS, only one stimulated the posterior parietal cortex in patients in MCS or with unresponsive wakefulness syndrome (UWS) [15], with the rest of the studies stimulating the left DLPFC and none employing right anodal tDCS in patients with DOC [6–8, 16–18]. The right dorsolateral frontal cortex probably exerts top-down control over noradrenergic activation and is linked to the reticular structure of the brainstem [19, 20], which is essential for the maintenance of wakefulness. Moreover, the right hemisphere has dominance for attention [21]. Our previous functional MRI (fMRI) findings showed that the right DLPFC might be essential for consciousness improvements [22]. Furthermore, targeting the right DLPFC with high frequency repetitive TMS has been shown to effectively improve consciousness in patients with DOC [23, 24]. Hence, we hypothesize that tDCS stimulating the right DLPFC could be an effective noninvasive technique for treating DOC.

In conclusion, this study had three main objectives. The first one was to determine whether repeated tDCS of the left or right DLPFC was safe for DOC patients by evaluating any adverse effects. The second one was to determine whether repeated tDCS targeting the DLPFC had an effect on DOC patients by tracking changes in behavioral and EEG performance after a two-week course of tDCS and thereafter performing long-term outcome evaluations 3 months later. To better characterize the underlying neural mechanisms, we used graph-theoretic approaches with EEG to investigate changes in tDCS-related functional connectivity. The third one was to determine the more appropriate target for tDCS between the left and the right DLPFC. Therefore, we designed a double-blind, randomized, sham-controlled study to primarily investigate and compare the efficacy of left anodal tDCS, right anodal tDCS, and sham stimulation setups.

## 2. Materials and Methods

### 2.1. Patients

**Figure 1:**
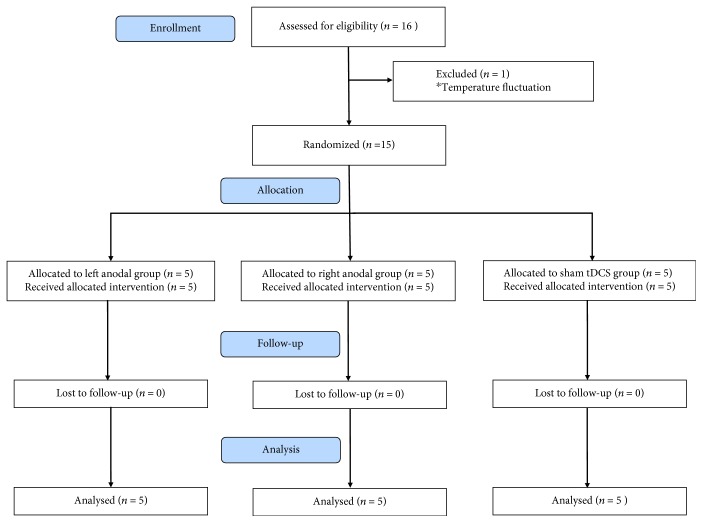
CONSORT flow diagram.

A convenience sample of 16 patients in the Department of Rehabilitation at Hangzhou Hospital of Zhejiang CAPR was included in the prospective study. All subjects met the study inclusion criteria: (1) no use of centrally acting drugs, (2) no use of neuromuscular function blockers and no sedation within 24 hours prior to the study, (3) periods of spontaneous eye opening (indicating preserved sleep-wake cycles), and (4) a diagnosis of UWS or MCS based on Coma Recovery Scale-Revised (CRS-R) assessment [25]. Participants with a history of epilepsy or who had a metallic cerebral implant or pacemaker were excluded [16]. Before stimulation, patients underwent a 1-week screening, which included behavioral assessment and routine laboratory studies, to assess the stability of each patient's condition. After this screening process, we excluded one patient due to temperature fluctuation (Figure 1). Demographic and clinical characteristics of the enrolled patients are shown in Table 1.

The trial was conducted in accordance with the Declaration of Helsinki. Written informed consent was obtained by the legal representative of each patient. This study was approved by the Ethical Committee of the First Affiliated Hospital, School of Medicine, Zhejiang University and Hangzhou Hospital of Zhejiang CAPR.

### 2.2. Study Overview

**Figure 2:**
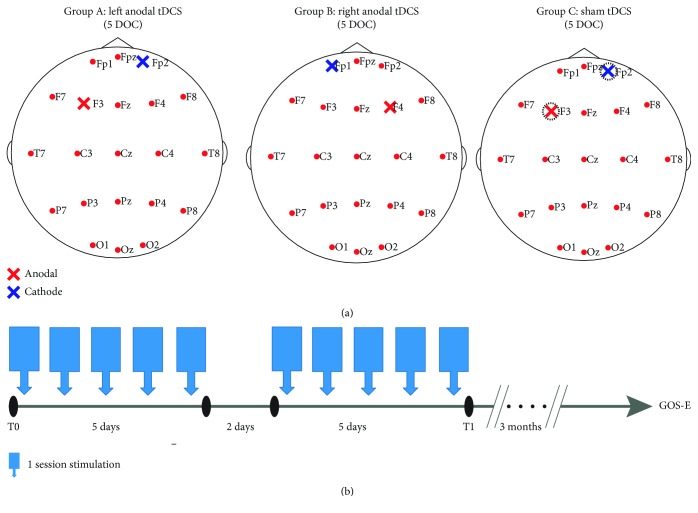
Overview of experimental structure. (a) 15 patients were equally divided into 3 groups as group A (left anodal tDCS), group B (right anodal tDCS), and group C (sham tDCS). Stimulation sites of each group were marked on the international 10–20 system. The anode is colored red, the cathode blue. (b) Stimulation protocol. One session per day, for a total of 10 sessions in a 2-week period. Evaluations were performed at baseline (T0), immediately after stimulation (T1), and 3 months later.

The study had three phases. The first one was a 1-week screening of the patients. This was followed by a 2-week treatment phase, which involved daily treatment with active or sham tDCS. Fifteen patients were randomized at a ratio of 1 : 1 : 1 to each treatment group (groups A and B as the experimental groups and group C as the control group). During the 2-week acute treatment phase, tDCS sessions were scheduled daily for a total of 10 sessions. Moreover, all patients underwent behavioral and EEG evaluations at the start and end of the 2-week treatment. Finally, after the tDCS treatment, each patient returned for a 3-month follow-up to check the outcome. Throughout the study, patients were required to maintain their original medication regimen. The research protocol is shown in Figure 2.

### 2.3. Stimulation Protocol

tDCS was administered using a DC-stimulator (neuroConn GmbH, Ilmenau, Germany). In group A, direct current was applied using a battery-driven constant-current stimulator through saline-soaked surface sponge electrodes (7 cm × 5 cm) with the anode placed over the left DLPFC (F3 according to the 10-20 international EEG system) and the reference cathode placed over the right supraorbital region (Fp2) [16]. In group B, the anode was placed over the right DLPFC (F4) and the cathode placed over the corresponding contralateral supraorbital area (Fp1) [26, 27]. During the real tDCS (groups A and B), the current was increased to 2 mA at the onset of stimulation for 20 min per session. It was administered once a day, for 10 working days (from Monday to Friday in two consecutive weeks). For the sham tDCS, the same stimulation parameters were employed, except that the stimulator had a built-in placebo mode; when it was activated, two ramp fade-in/fade-out periods at the beginning and the end of sham stimulation mimicked the somatosensory artifact of real tDCS [12]. The electrodes were placed on the scalp for 20 min as in the other groups, with the anode and cathode being placed over the left DLPFC (F3) and right supraorbital region (Fp2), respectively. It was also administered for 10 working days.

### 2.4. Safety and Behavior Assessment

All patients were assessed by two trained and experienced blinded hospital staff using the CRS-R at study entry and after the tDCS sessions. For baseline assessment, 2 blinded assessors independently performed CRS-R assessments in a randomized order, thus permitting interrater comparisons. The CRS-R consists of 23 hierarchically arranged items that comprise 6 subscales addressing auditory, visual, motor, verbal, communication, and arousal functions [25]. After each session, the staff completed a safety assessment. Skin redness, electrolytic burns, excessive sweating, and epilepsy were regarded as adverse effects.

The long-term outcome was assessed 3 months after the trial using the Glasgow Outcome Scale-Extended (GOS-E). A GOS − E value < 4 was termed as “outcome-negative,” while a GOS − E value ≥ 4 was considered as “outcome-positive” [16, 28].

### 2.5. EEG Data Collection and Preprocessing

For each participant, we collected 64-channel EEG data with a BrainAmp (Brain Products, Gilching, Germany) amplifier for at least 15 minutes at a 1000 Hz sampling rate. The impedance of all the electrodes was kept below 5 k*Ω*. Scalp electrodes were positioned according to the international 10–20 system. All electrode sites were referenced online to FCz.

EEG data were analysed offline using the EEGLAB toolbox and processed using an average reference. After offline referencing, the EEG signal was high-pass filtered at 0.1 Hz and low-pass filtered at 30 Hz and then segmented into 3-second-long epochs. Each epoch was baseline corrected relative to the mean voltage over the entire epoch. The artifact-free periods were submitted to independent component analysis (ICA) using the runica function, and the bad channels were interpolated using the planar gradiometers incorporated in EEGLAB.

### 2.6. EEG Functional Connectivity

Generally speaking, phase synchronization is often calculated from the phase or the imaginary component of the complex cross-spectrum between the signals measured at a pair of channels [29]. For example, phase locking value (PLV) is a well-known EEG measure of information exchange between neuronal populations. To lower the effect of volume conduction, 21 canonical electrodes of the 10–20 system were selected from a total of 64 electrodes to construct the brain network [30]. The described network analysis procedure was performed for the 3 s long segment of each subject, resulting in one connectivity (adjacency) matrix with a dimension of 21 × 21 for each subject. Based on the connectivity matrix, the network topology was calculated for each subject in different frequency bands (delta, 0.1-4 Hz; theta, 4-8 Hz; alpha, 8-13 Hz; and beta, 13-30 Hz).

Here, let *t* denote the sampling period and *N* denote the sample number; for two time series, *x*(*t*) and *y*(*t*), the instantaneous phases are *ɸx*(*t*) and *ɸy*(*t*), respectively, and the PLV is formulated as:

(1) $$\begin{array}{c} plv=\left|\frac{1}{N}\overset{N-1}{\underset{j=0}{\sum }}e^{i\left(\phi_{x}\left(jt\right)-\phi_{y}\left(jt\right)\right)}\right| \end{array}$$

For each subject, the final weighted brain network, an adjacency matrix with dimension of 21 × 21, was obtained by averaging matrices of those artifact-free data segments. Specifically, the whole brain was divided into five cortical regions: left frontal, right frontal, central, parietal, and occipital lobes; we sought to characterize the effects of tDCS on functional connectivity between brain regions.

### 2.7. Statistics Analysis

One-way ANOVA and Fisher's test were used to compare the continuous and categorical variables among the three groups, respectively. The CRS-R total and subscale scores were also separately evaluated using the repeated measures ANOVA with “Protocol” as between-subject factors and “Time” as within-subject factors. When statistically significant differences (*α* = 0.05) were found in the main effects, post hoc Bonferroni corrections for multiple comparisons were performed; while the interaction effect was significant, simple effects tests were followed. GOS-E scores were analysed by the one-way ANOVA to compare differences in outcome between various stimulation conditions. The sphericity assumption was assessed using Mauchly's test before conducting repeated measures ANOVA. When the assumption was rejected, the Greenhouse-Geisser correction was used to adjust the degrees of freedom.

## 3. Results

As presented in Figure 1, out of the 16 eligible patients, one patient was excluded because of temperature fluctuations. The remaining 15 patients were assigned to receive tDCS. During the period of tDCS treatment and at the 3-month follow-up, no patient withdrew from the study. The three classified groups did not differ in diagnosis, time since onset, age, sex, or etiologies (*P* > 0.05). Overall, no side effects were observed in each group.

### 3.1. CRS-R Scores

At the individual level, only 2 patients (P2 and P3) in group A showed an increase in the CRS-R scores that was not associated with changes in clinical diagnosis, from 8 to 15 points for P2 and from 8 to 10 points for P3, after application of anodal tDCS on the left DLPFC. For P2, the CRS-R-visual scoring improved prominently by acquiring the ability of object localization; moreover, auditory, motor, and communication ability also improved marginally even though the patient remained in MCS. For P3, the CRS-R-auditory scoring increased. This was determined by occurrence of an eyelid flutter or eye blink immediately after noise stimulus. Surprisingly, all five individuals in the right-anode tDCS group showed no detectable behavioral modification. Likewise, all five patients under the sham stimulation demonstrated no improved behavioral performance. P11 and P15 remained in UWS with a CRS-R score of 4 while P12-P14 remained in MCS (the CRS-R scores were 9, 12, and 10, respectively). One-way ANOVA showed that there was no significant difference in the CRS-R total scores as well as the six subscales between the groups at baseline (T0). In addition, repeated measures ANOVA did not show any significant difference in the time (T0, T1), protocol (left-anode, right-anode, and sham), interactions (*P* > 0.05), or the main effect of time or protocol (*P* > 0.05) on either the CRS-R total or the six subscores.

### 3.2. EEG Functional Connectivity

**Figure 3:**
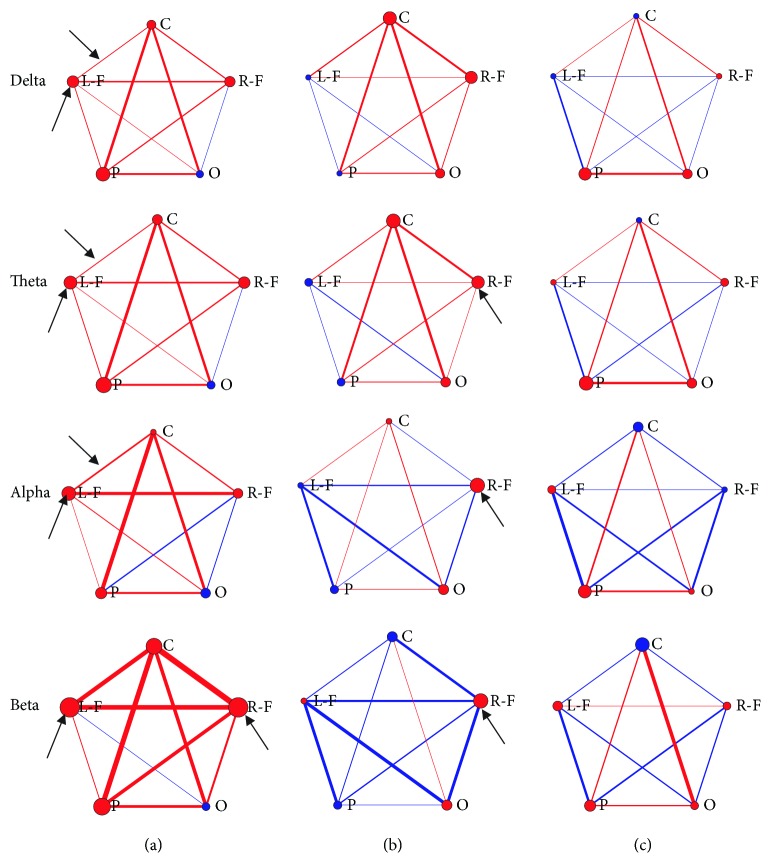
Connectivity changes between interest brain regions with tDCS. (a) An enhancement of a broadly anatomically distributed network with left anodal tDCS. (b) Increased delta-/theta-band coherence occurred after right anodal tDCS. (c) No significant effects observed in the sham group. The increased (red) or decreased (blue) proportion change between the two brain regions was indicated by lines and filled circles. The bigger the circle (or the thicker the line), the larger the modified connection. The statistically significant changes were marked with arrows (*P* < 0.05, paired *t*-test).

The averaged network connectivity in each group is listed in Figure 3. Left anodal tDCS increased connectivity between the left frontal lobe and several regions of the cortex, such as the right frontal, central, and parietal cortexes. Evidently, the internal connection of the left frontal lobe was the most salient among the four frequency bands (Figure 3(a)). When the right DLPFC was stimulated, the delta and theta connectivity in the widespread cortex was enhanced, whereas the high-frequency band (alpha and beta) oscillation only increased in the right frontal lobe (Figure 3(b)). In contrast, Figure 3(c) shows a minimal increase in connectivity after the sham tDCS, marked by thin red lines.

To ensure that the observed effects were not driven by the activity in a single subject and also because of the limited sample size, we analysed each subject's activity individually. The analysis was critical and rigorous to ensure that there was a high consistency in the direction of the functional connectivity changes (i.e., increase or decrease) in the majority of patients in the same group [31]. Moreover, statistics on changes in functional connectivity in regions of interest, such as the left frontal, right frontal, central, parietal, and occipital lobes were tested (pre- and posttreatment, paired *t*-test).

Specifically, high consistency was observed in four different frequency bands. There was a significant connectivity increase in the left frontal lobe. Stronger short connection patterns were observed in the left-frontal-central cortex in the left anodal tDCS group at delta, theta, and alpha frequencies, while a significantly increased beta oscillation was detected in the right frontal lobe (*P* < 0.05, marked with arrows in Figure 3(a)). No region-to-region connection showed a significant increase after right tDCS; however, the connections within the right frontal lobe were activated (*P* < 0.05, marked with arrows in Figure 3(b)). By contrast, all band coherences after sham stimulation did not increase significantly in pairings with electrodes of all the interest regions in the DOC patients (*P* > 0.05, Figure 3(c)).

**Figure 4:**
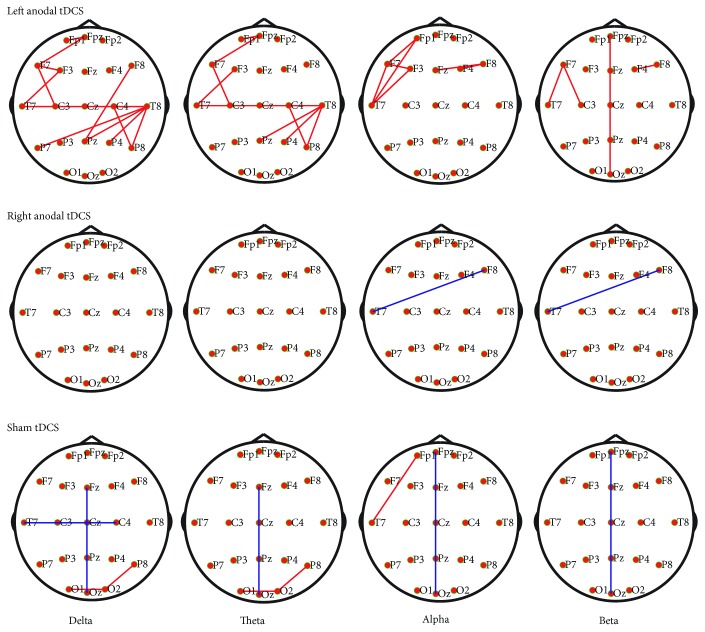
Network topography shows altered connectivity in four bands (columns) for the three tDCS groups (rows). Only the left anodal tDCS activated the brain network (*P* < 0.05, FDR corrected). Red lines mean significantly increased connectivity, and blue lines mean decreased connectivity.

**Figure 5:**
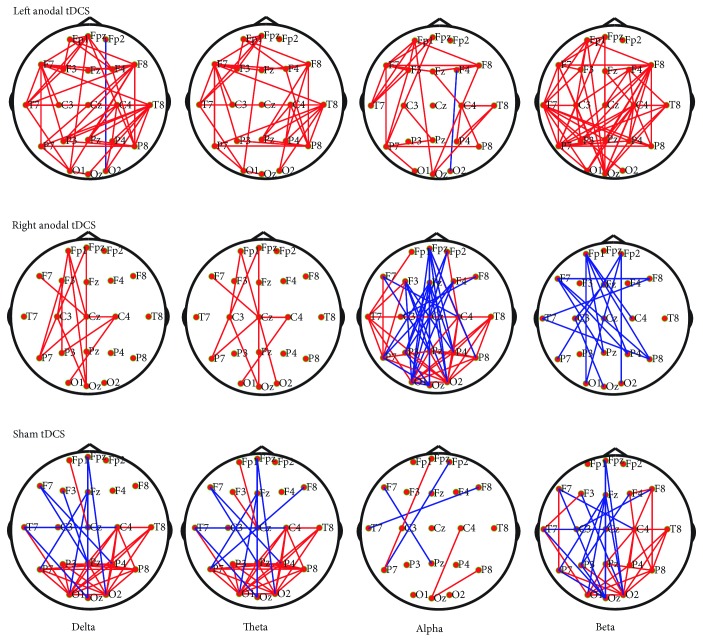
Significantly changed network patterns without FDR correction. The whole-brain cortex showed broadly enhanced connectivity after left DLPFC stimulation. Red lines mean significantly increased connectivity, and blue lines mean decreased connectivity (*P* < 0.05, uncorrected).

Next, to explore the effects of tDCS on the global cerebral cortex, we developed whole-brain connectivity topographies for the three groups. Pairwise comparisons of each connectivity were performed using paired *t*-tests, and correction of the false discovery rate (FDR) was performed after multiple comparisons (Figure 4). Only several increased connectivities were detected after left anodal tDCS (*P* < 0.05, FDR correction). Moreover, we also focused on the activation patterns without FDR correction. Visually, during left tDCS, the whole-brain cortex showed broadly enhanced connectivity, while during right tDCS, only the mesial frontoparietal areas, in low frequency, and the bilateral parietal-occipital areas showed significantly increased functional connectivity. However, no increased connectivity within the frontoparietal cortical area was observed after sham tDCS (Figure 5).

### 3.3. GOS-E

The long-term outcomes of the patients are shown in Table 1. Only one patient (P2) had positive outcomes (regained the ability to live independently). At the group level, no significant differences emerged between the real or sham stimulation conditions in GOS-E scores at T1 (*P* = 0.624).

## 4. Discussion

We conducted a randomized, sham-controlled tDCS clinical study on DOC patients. Anodal tDCS applied over the left or right DLPFC for two weeks was well tolerated, and the right anodal protocol was, for the first time, certified as a safe paradigm for DOC. Despite the absence of significant behavioral changes after group analysis, a trend toward higher functional connectivity was observed in the DOC patients after 2 weeks of tDCS of both the left and right DLPFC compared to sham stimulation; however, the detectable EEG connectivity changes were only observed close to the anode stimulation sites, but not in distant brain regions.

For patient 2, the clinical characteristics might have contributed to the recovery. It is well-known that traumatic patients usually recover better than do patients with anoxic injury, with recovery occurring within the first few months of injury. The behavioral modifications observed are similar to those observed in previous studies. Estraneo et al. reported moderate improvements after 5 days of tDCS with the anode positioned over the left DLPFC, with some patients progressing to a better clinical condition with substantial behavioral change during the systematic follow-up period [6]. Another study reported long-term effects in the 12-month follow-up rather than immediate effects after 10 tDCS sessions in two weeks [17]. In our study, one patient showed a positive outcome after assessment using GOS-E. There is evidence showing that neuroplasticity changes after application of noninvasive brain stimulation protocols do not necessarily appear immediately after the stimulation but may arise after a temporal delay [13, 32]. Nonetheless, it cannot be definitively concluded that the tDCS effects can be maintained until 3 months since the spontaneous recovery of the patients cannot be excluded. Further studies with larger sample sizes are needed to explore the long-term effects of tDCS. On the other hand, initial or acute changes in cortical activity may not cause a cascade of coactivation. Notably, the longer delay observed in the stimulation of the patients' cortical activity should not be ignored, and further studies should track dynamic changes in functional connectivity after tDCS over a long-term period. However, our findings differ from those of several previous studies on tDCS that reported positive results. Zhang et al. reported clinically significant improvement in all MCS patients and 2 out of 5 UWS patients after two tDCS per day for 10 consecutive working days [33]; this difference in results may be due to the number of stimulation sessions because we only performed one tDCS per day.

Studies in healthy patients have shown that prefrontal tDCS influences coactivation in the frontal parts of the default mode network, as well as the frontal-parietal network. Functionally, an increased coactivation of the frontal and parietal regions has been related to top-down modulation of cognitive ability [12]. Therefore, the lack of top-down modulation after tDCS may fail to enhance the state of alertness and hence affect alertness-dependent cognitive functions [34], which is also consistent with lack of CRS-R improvement. In addition, based on neuroimaging studies, tDCS seems to influence the activity of not only the stimulated area but also the brain network connectivity encompassing long-distance brain areas [11, 13]. Some studies on DOC have reported portions of long-distance connectivity encompassing cortical or subcortical regions as chronically underactive or underlying a breakdown [35–37]; hence, the action of tDCS on network connectivity may be counteracted, thus fading the propagation of tDCS activity [38]. Particularly, the enrolled DOC patients in our study were in poor condition with low CRS-R scores at baseline, which indicate more severe impairment of functional interregional connectivity based on the correlation between behaviorally defined clinical entities and the underlying brain damage [39]. In this vein, the severity of brain damage may be related to the effectiveness of tDCS.

Moreover, independent of the variability of the cortical damage, residual brain activity in the stimulated area is necessary for effective stimulation, as shown by a study on patients with stroke that illustrated that the effects of tDCS on the motor area were limited when the pyramidal tract was damaged [40]. Moreover, another retrospective study also reported that MCS unresponsive to tDCS showed hypometabolism in the DLPFC [41]. In the current study, it is possible that there was inhibition of the left or right prefrontal metabolism based on existing neuroimaging and PET studies performed on MCS and UWS [42–44]. Further studies on tDCS of DOC patients should investigate the residual regional brain metabolism and cortical connectivity to determine if there is preservation of the prefrontal cortex, which might provide important information to inform guidelines on the administration of tDCS to DOC patients.

Previous studies have applied tDCS over the left/right DLPFC and obtained empirical evidence from observations of modulation effects that point toward hemispheric lateralization of emotional processing, decision-making, response selection, and attention [45–47]. Taken together, these results emphasize the presence of hemispheric asymmetry and a facilitating effect of anodal stimulation of the left DLPFC on neuronal activity. Stimulation of the left DLPFC apparently has a larger beneficial impact on neural activity than does sham or right DLPFC stimulation, underscoring the important roles of this prefrontal brain region and its related network. Similarly, our findings also revealed stronger effects after stimulation of the left DLPFC. We speculate that the facilitation effect on the left or right DLPFC might involve several pathways. The specific left stimulation protocol might influence wider corticocortical and corticothalamic connectivity, which are severely degenerated in patients with DOC [39]. However, future studies are needed to validate the extent to which roles in consciousness and cognition are differentially assigned between the left and the right DLPFC.

Our study has some limitations. Considering the scarcity of the CRS-R and behavioral fluctuations of these patients, identification errors might exist. Before the stimulation, we performed a 5-day behavioral assessment; however, we only assessed behavioral performance once with two staff members after the 2-week treatment phase. Performing CRS-R assessments daily has been suggested to reduce assessment errors and also to allow dynamic assessment of tDCS efficiency. Moreover, this study conducted a solely unilateral tDCS protocol due to the limited number of patients. Recently, a new tDCS electrode arrangement with bilateral tDCS was shown to yield prominent behavioral modification in healthy subjects and chronic stroke patients [13, 48]. The superiority of bilateral tDCS over unilateral tDCS has been assumed to be related to a more pronounced interference of interhemispheric information processing and more widespread connectivity changes [13]. In addition, a single session of multitarget tDCS induced larger effects in patients with Parkinson's disease than in those induced by M1 stimulation alone [3]. Similarly, simultaneous facilitation of motor and cognitive mediators might induce a greater beneficial impact in patients with DOC, given their characteristic motor and cognitive disturbances. So far, the efficacy of all types of brain stimulations in DOC patients is still unclear [49]. There is still a need for further investigation and exploration of better stimulation patterns. Another limiting factor of the current study is that the small sample size may have reduced statistical power. Although individual analysis was performed in the current study, more studies with larger sample sizes are needed to validate the electrophysiological effects of tDCS on patients with DOC. However, this study provides novel evidence for the safety of tDCS of the right DLPFC. However, this evidence should be interpreted cautiously, and additional research is needed to understand the underlying mechanisms of different interventions for ameliorating unconsciousness.

## 5. Conclusions

The left tDCS design facilitated the excitability of the prefrontal cortex. Targeting the right DLPFC was less effective and merely led to activation of the stimulation sites. These results provided evidence for a hemispheric asymmetry of tDCS effects in patients with DOC. However, our findings are preliminary, and future studies with large sample sizes are needed to support these findings.

## Figures and Tables

**Table 1:** Clinical characteristics and behavioral assessment of DOC patients.

Patient ID	Clinical diagnosis	Gender/age	Etiology	Month since injury	CRS-R scores at T0	CRS-R scores at T1	GOS-E	Outcomes
01	MCS	M/77	Hemorrhage	201	13 (2/3/3/1/1/3)	13 (2/3/3/1/1/3)	2	Negative
02	MCS	M/16	Trauma	42	8 (1/1/3/1/0/2)	15 (2/4/5/1/1/2)	7	Positive
03	MCS	M/16	Hemorrhage	283	8 (1/3/1/1/0/2)	10 (2/3/1/1/0/3)	2	Negative
04	UWS	F/57	Trauma	168	5 (0/1/1/1/0/2)	5 (0/1/1/1/0/2)	2	Negative
05	UWS	M/56	Hemorrhage	68	5 (1/0/2/0/0/2)	5 (1/0/2/0/0/2)	3	Negative
06	UWS	F/58	Hemorrhage	158	3 (0/0/1/0/0/2)	3 (0/0/1/0/0/2)	2	Negative
07	UWS	M/67	Hemorrhage	55	2 (0/0/0/0/0/2)	2 (0/0/0/0/0/2)	2	Negative
08	UWS	F/40	Hemorrhage	76	6 (0/1/2/1/0/2)	6 (0/1/2/1/0/2)	3	Negative
09	MCS	M/43	Hemorrhage	631	11 (3/2/2/1/0/3)	11 (3/2/2/1/0/3)	3	Negative
10	UWS	F/66	Trauma	219	7 (1/1/2/1/0/2)	7 (1/1/2/1/0/2)	2	Negative
11	UWS	M/37	Anoxia	55	4 (1/0/1/0/0/2)	4 (1/0/2/1/0/2)	2	Negative
12	MCS	F/59	Anoxia	87	9 (1/2/2/2/0/2)	9 (1/2/2/2/0/2)	3	Negative
13	MCS	M/34	Trauma	174	12 (2/3/2/2/1/2)	12 (2/3/2/2/1/2)	2	Negative
14	MCS	F/53	Anoxia	54	10 (2/2/3/1/0/2)	10 (2/2/3/1/0/2)	3	Negative
15	UWS	M/39	Trauma	21	4 (0/0/2/0/0/2)	4 (0/0/2/0/0/2)	3	Negative
16	UWS	M/44	Trauma	98	3 (1/1/1/0/0/2)	/	/	/
*P*	0.343	0.435/0.537	0.741	0.327	0.593	0.346	0.624	/

Note: P1-P5 were in the left-anodal group, P6-P10 were in the right-anodal group, P11-P15 were in the sham group, and P16 was excluded due to unstable state. T0: at baseline; T1: immediately after 2-week stimulation.

## Data Availability

The data used to support the findings of this study are available from the corresponding author upon request.

## Abbreviations

L-F: left frontal
R-F: right frontal
C: central
P: parietal
O: occipital.
